# First report on antibiotic resistance and antimicrobial activity of bacterial isolates from 13,000-year old cave ice core

**DOI:** 10.1038/s41598-020-79754-5

**Published:** 2021-01-12

**Authors:** Victoria I. Paun, Paris Lavin, Mariana C. Chifiriuc, Cristina Purcarea

**Affiliations:** 1 Department of Microbiology, Institute of Biology, Bucharest, Romania; 2 The Network for Extreme Environment Research (NEXER), Scientific, Universidad de Antofagasta, Antofagasta, Chile; 3 Departamento de Biotecnología, Facultad de Ciencias del Mar y Recursos Biológicos, Universidad de Antofagasta, Antofagasta, Chile; 4 Faculty of Biology and the Research Institute of the University of Bucharest, ICUB, University of Bucharest, Bucharest, Romania

**Keywords:** Microbiology, Biogeochemistry, Climate sciences, Ecology, Environmental sciences

## Abstract

Despite the unique physiology and metabolic pathways of microbiomes from cold environments providing key evolutionary insights and promising leads for discovering new bioactive compounds, cultivable bacteria entrapped in perennial ice from caves remained a largely unexplored life system. In this context, we obtained and characterized bacterial strains from 13,000-years old ice core of Scarisoara Ice Cave, providing first isolates from perennial ice accumulated in caves since Late Glacial, and first culture-based evidences of bacterial resistome and antimicrobial compounds production. The 68 bacterial isolates belonged to 4 phyla, 34 genera and 56 species, with 17 strains representing putative new taxa. The Gram-negative cave bacteria (Proteobacteria and Bacteroidetes) were more resistant to the great majority of antibiotic classes than the Gram-positive ones (Actinobacteria, Firmicutes). More than 50% of the strains exhibited high resistance to 17 classes of antibiotics. Some of the isolates inhibited the growth of clinically important Gram-positive and Gram-negative resistant strains and revealed metabolic features with applicative potential. The current report on bacterial strains from millennia-old cave ice revealed promising candidates for studying the evolution of environmental resistome and for obtaining new active biomolecules for fighting the antibiotics crisis, and valuable cold-active biocatalysts.

## Introduction

Earth is a primarily cold planet, with more than 85% of the biosphere being exposed to permanent temperatures below 5 °C and with frozen habitats covering up 20% of our planet surface^1,2^. Thus, cold-adapted microbiomes are very important for the global ecology, while their biological activities maintain the nutrient flux in the environment and contribute to the global biogeochemical cycles, being also sensitive indicators of climate changes^3^. Moreover, their unique physiology and metabolic pathways provide essential knowledge for understanding the adaptation mechanisms to low temperatures and a valuable source of biomolecules for biotechnological applications. To overcome the challenges of life in harsh conditions (nutrients scarcity, high salinity, dryness, and low water activity, high UV irradiation and oxidative stress at high altitudes, and high pressure in the deep sea)^4^, cold-adapted microorganisms have developed a wide range of adaptations, from cellular envelope (incorporation of unsaturated membrane fatty acids and carotenoid pigments, cell wall peptidoglycan layer, modified lipopolysaccharides of the outer cell membrane) and metabolic adaptation (antifreeze and ice-nucleating proteins, cold-active enzymes with increased structural flexibility, cold-inducible promotors, increased variety and number of tRNA species, multiple stress-responsive genes), to cryoprotectants (extracellular polymeric substances, biosurfactants, compatible solutes) and increased variety and number of chaperons production, and novel metabolic capabilities (microbial growth and multiplication possible at 0–30 °C, vitrification, storage materials such as polyhydroxyalkanoates and cyanophycins), that can be exploited to develop novel biotechnological perspectives^5^. Despite the constantly increasing data on the microbial composition and diversity in Polar ice sheets and glaciers^6–9^, Polar soil and permafrost^10,11^, and aquatic environments^12,13^, microbiomes entrapped in perennial ice from caves still remain a largely undiscovered life system^14^.

Among the explored ice caves, Scarisoara Ice Cave (Romania) constitutes a unique cold environment, its 100,000 m^3^ perennial ice block dated about 13,000-years before present (BP) being one of the oldest and largest in the world^15–18^.

Recent investigations revealed the presence of cultivable bacteria^19–22^ and fungi^23,24^ in cave ice deposits. The microbial diversity and geochemical dependence of uncultured microbiome from underground frozen habitats highlighted complex bacterial communities in sediments from Antarctic volcanic ice caves from Mount Erebus^25^, and both sediments and ice from Hawaiian lava tubes^26^. A rich uncultured prokaryotic diversity was also found in Scarisoara cave ice strata of different ages^18,27,28^, in addition to a complex fungal community^29^. The distribution of the total and putatively active bacterial and fungal communities appeared to be modeled by the climatic characteristics during ice deposition and the organic carbon content of the ice substrate^18,22,24,28,29^.

Antimicrobial resistance (AR) is nowadays in top ten of global threats, concerning the animal and human health, food security and affecting the entire societal development (WHO 2020, www.who.int)^30^. AR is an old natural phenomenon, the antibiotic biosynthetic pathways and the resistance genes evolving over millions of years^31–35^. More evidence indicated environmental resistome as the reservoir for resistance genes found in clinical pathogens^36,37^. Due to their multiple physiological roles in natural environments, antibiotics acted as selecting and accelerating factors of AR evolution^32,38,39^. Bacterial strains highly resistant to known antimicrobial compounds usually have the potential for antimicrobial activity against other bacteria^9^.

Taking into account that extreme environments mirror a very remote moment from our planet past, prospecting their microbiome could provide a reference time point and new data for understanding the AR occurrence and evolution, and promising leads for new biologically active compounds including antimicrobials^40–43^. Cold-adapted microbes isolated from Polar habitats, mostly Actinobacteria and Proteobacteria from various Antarctic and Arctic habitats demonstrated their ability to produce antimicrobial compounds and activity against human pathogens^9,44–48^. However, due to the rather scarce recovery of psychrophilic and psychrotrophic antibiotic producers from cold environments^43^, limited information on the antimicrobial susceptibility is available as compared to that of mesophiles^49^.

Most studies on bacterial communities found in ice caves—unique, secluded, light-deprived and low content nutrients icy habitats—mainly focused on the identification and characterization of their diversity and composition and their response to climatic and environmental parameters during ice deposition^14^. So far, viable bacterial communities from Scarisoara cave ice were reported in distinct ice strata formed during the last 900 years by PCR-DGGE screening of the ice microbial cultures^22^. In this context, our investigation focused on isolating bacterial strains from up to 13,000-years old cave ice core, providing first cave isolates from perennial ice deposited since the Late Glacial period, and first culture-based evidences of cave ice bacteria resistome and their ability to produce antimicrobial compounds, representing putative novel tools to fight antibiotic resistance. In addition, screening for various biochemical features of cave ice isolates revealed a new reservoir of cold-active enzymes as valuable candidates for industrial applications.

## Results

### Bacterial strains isolated from the ice chronosequence of Scarisoara Ice Cave

A total of 68 distinct bacterial strains were isolated from the 13,000-years old ice core of Scarisoara cave by cultivation at 4 °C and 15 °C on R2A medium (Supplementary Table S1). These isolates retrieved from 19 cave ice samples of the ice chronosequence at an interval of ~ 300 years covered all ice layers, with a higher number originating from the 10,000-years old (9 strains), and 400-, 1000- and 7000-years old ice (7 strains) ice strata (Supplementary Table S2). Amongst strains with identical 16S rRNA amplicon sequence, SC51B.2 and SC14F.2 exhibited different growth temperature intervals (Supplementary Table S1), and *Microbacterium hydrocarbonoxydans*, *Microbacterium pygmaeum* and *Chryseobacterium hominis* isolates differed by their antibiotic susceptibility profiles (Supplementary Table S3).

**Figure 1:**
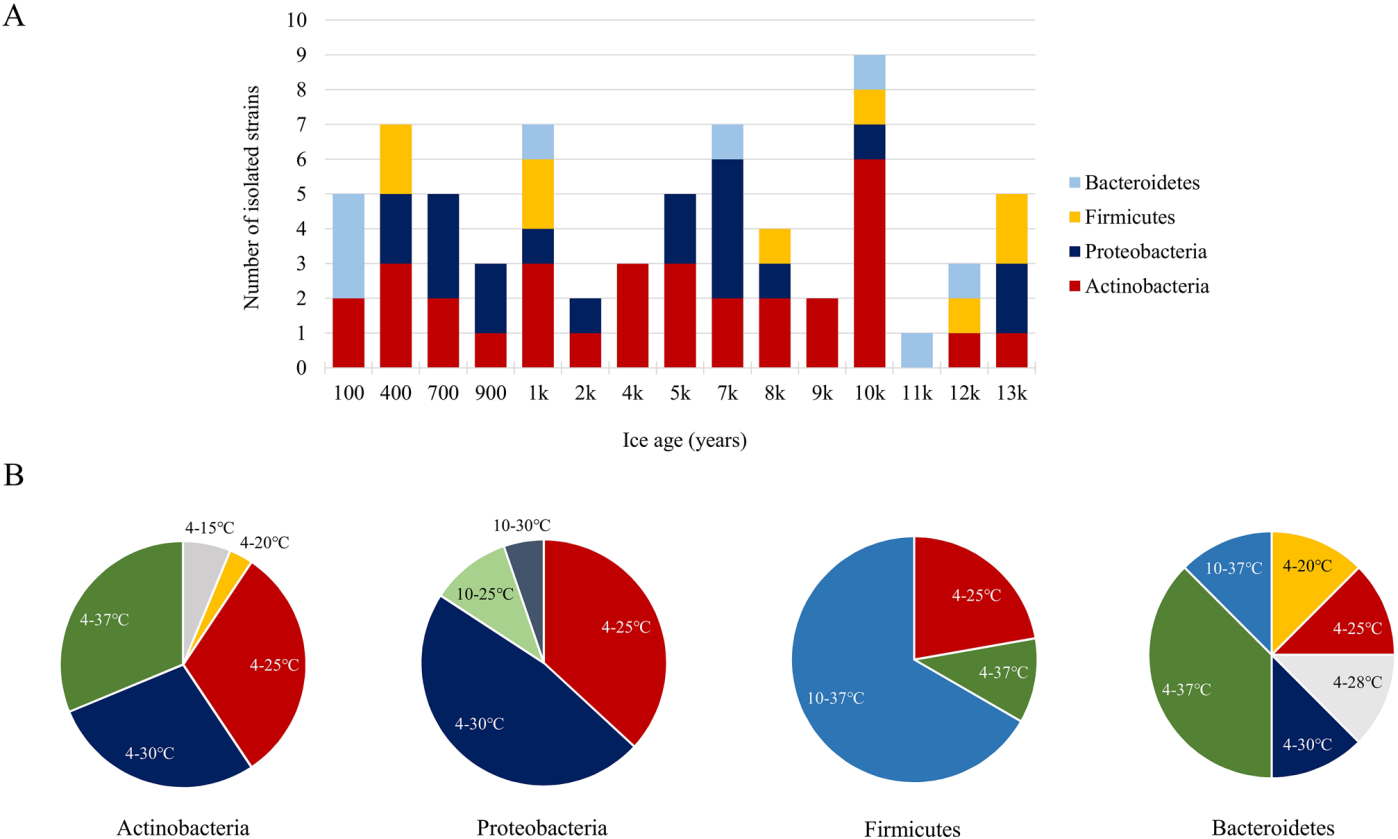
Phyla and growth temperature distribution of cave ice bacterial isolates along Scarisoara ice core. (**A**) Number of phyla in ice core strata up to 13,000 years BP; (**B**) Growth temperature interval distribution of cave isolates belonging to Actinobacteria, Proteobacteria, Firmicutes and Bacteroidetes phyla.

The isolates were assigned to 4 phyla, 34 genera and 56 species, based on a 16S rRNA gene sequence identity of 89–100%, with 535–1299 bp amplicon coverage (Supplementary Table S1). Molecular identification revealed various phylogenetic groups belonging to phyla Actinobacteria (32 strains), Proteobacteria (19 strains), Firmicutes (9 strains) and Bacteroidetes (8 strains) (Supplementary Table S1). Actinobacteria were present in all ice layers except for 11,000-years old ice, Proteobacteria species in 11 out of 19 strata, while Firmicutes and Bacteroidetes in only 6 and 5 layers, respectively (Fig. 1A).

Most of Actinobacteria isolates belonged to family Microbacteriaceae (22 strains), in addition to Micrococcaceae (6 strains), Nocardioidaceae (3 strains) and Dietziaceae (1 strain) (Supplementary Table S1). Proteobacteria isolates mainly belonged to class Aphaproteobacteria (10 strains), with species of famillies Caulobacteraceae (6), Phyllobacteriaceae (2) and Sphingomonadaceae (2). Betaproteobacteria isolated strains were assigned to Alcaligenaceae (5) and Comamonadaceae (1) families. The Gammaproteobacteria strains (3) belonged to order Pseudomonadales. Phylum Firmicutes was represented by Bacillaceae (5 strains), Planococcaceae (3 strains) and Paenibacillaceae (1 strain) families. Bacteroidetes isolates were assigned to families Flavobacteriaceae (5 strains) and Sphingobacteriaceae (3 strains).

At genus level, a high majority of cave strains were singular, except for *Microbacterium* (13), *Bacillus* (5), *Arthrobacter* (4), *Brevundimonas* (4), *Chryseobacterium* (4), *Cryobacterium* (3), and 2 isolates of *Aeromicrobium*, *Paralcaligenes*, *Pedobacter*, *Phyllobacterium*, *Pseudarthrobacter*, *Pseudomonas* and *Sporosarcina* genera (Supplementary Table S1).

Among the 56 identified species, a high number belonged to Actinobacteria (24) and Proteobacteria (19), with a lower representation of Firmicutes (7) and Bacteroidetes (6) (Supplementary Table S1). Most species were singular, only 9 being found in different ice strata. The widest distribution along the ice core was observed for *Microbacterium hydrocarbonoxydans* present in 100, 400, 900, 5000, 8000 and 12,000-years old ice (Supplementary Table S1), and *M. pygmaeum* originating from 700, 4000, 5000 and 9000-years old ice strata. *Chryseobacterium hominis* strains were isolated from old ice layers (7000, 10,000, and 11,000-years old ice). In addition, two different *Bacillus safensis* homologs were retrieved from old strata (12,000 and 13,000-years BP), and different *B. thuringiensis* strains from 400-years BP ice. The isolates from the oldest cave ice layers also comprised *Aeromicrobium panaciterrae, Paenibacillus amylolyticus, Pseudomonas brenneri,* and *P. grimontii* homologs among the Late Glacial cave ice bacterial strains (Supplementary Table S1).

The growth temperature interval of the cave isolates varied, with a minimum of 4 °C or 10 °C, and upper values ranging from 15 to 37 °C (Supplementary Table S1). The majority (58) of the strains had a minimum growth temperature of 4 °C, while 10 strains grew only above 10 °C (Supplementary Table S1; Fig. 1B). Based on this parameter^50^, four cave isolates were classified as psychrophiles, growing in the 4–15 °C interval (*S. xinjiangense* SC80A.3 and *Cryobacterium levicorallinum* SC21E.1), and 4–20 °C range (*Flavobacterium glaciei* SC1A.2 and *Arthrobacter* sp. SC41AB.3). The remaining 64 strains were psychrotrophs, with minimum growth temperatures of 4 °C and 10 °C. Among these, a variable number of Actinobacteria (10), Proteobacteria (7), Firmicutes (2) and Bacteroidetes (1) strains were cultivated between 4 and 25 °C, *Phenylobacterium* sp. SC71.1B and *Eoetvoesia caeni* SC41AB.2 could grow between 10 and 25 °C, and *Pedobacter bambusae* SC1A.4 within the 4–28 °C temperature interval (Supplementary Table S1; Fig. 1B). An upper limit for growth was accepted by the majority of isolates growing within 4–30 °C (19), 4–37 °C (14), 10–37 °C (7), most of them belonging to *Bacillus* spp*.*, and 10–30 °C (1) intervals (Supplementary Table S1; Fig. 1B).

Blast analysis of 16S rRNA gene fragments indicated that 11 of the isolates were homologous to cold-environments bacteria (Supplementary Table S1), including *Arthrobacter* (2 strains), *Cryobacterium* (3 strains), *Leifsonia* (1 strain), *Glaciihabitans* (1 strain), *Salinibacterium* (1 strain), *Psychrobacter* (1 strain), *Paenisporosarcina* (1 strain) and *Flavobacterium* (1 strain). Among these, *F. glaciei* SC1A.2, *S. xinjiangense* SC80A.3, and *C. levicorallinum* SC21E.1 were psychrophiles. The remaining 8 isolates (*A. psychrochitiniphilus* SC21C.1, *A. alpinus* SC86E.1, *Cryobacterium* sp. SC14E.1, *Leifsonia antarctica* SC7AB.2, *Glaciihabitans tibetensis* SC21E.3, *Psychrobacter glaciei* SC80A.2, *Cryobacterium flavum* SC83A.1 and *Paenisporosarcina macmurdoensis* SC80A.5) were classified as psychrotrophs based on their growth temperature range (Supplementary Table S1).

### Antibiotic susceptibility profile of cave isolates

Antimicrobial susceptibility of the cave isolates to 28 antibiotics of 17 classes having broad-spectrum, specificity for Gram-positive bacteria and for anaerobes revealed various resistance profiles (Supplementary Table S3). Gram-negative bacteria (Proteobacteria and Bacteroidetes) were more resistant to the great majority of antibiotic classes than the Gram-positive (Actinobacteria, Firmicutes) ones (Table 1). Both Gram-positive and Gram-negative isolates were highly resistant (> 77%) to metronidazole, mostly belonging to Bacteroidetes (100%), Proteobacteria (94.7%), and Actinobacteria (93.8%), while Firmicutes showed the lowest AR rate of 77.8%. Over 70% of phyla representatives were resistant to lincosamides and fatty acyls antibiotic classes (Table 1).

**Table 1:** Antimicrobial susceptibility of bacterial isolates from Scarisoara cave ice core.

Antimicrobial spectrum	Antibiotic class	Gram-positive bacteria (%)		Gram-negative bacteria (%)	
		Actinobacteria	Firmicutes	Proteobacteria	Bacteroidetes
Broad spectrum	Penicillins	37.5 ± 8.8	38.9 ± 7.9	76.3 ± 3.7	93.8 ± 8.8
	Cephalosporins	76.0 ± 17.2	79.6 ± 14.8	92.1 ± 7.3	95.8 ± 6.5
	Carbapenems	9.4 ± 0.0	0.0 ± 0.0	31.6 ± 0.0	62.5 ± 0.0
	Fluoroquinolones	67.2 ± 46.4	44.4 ± 15.7	84.2 ± 7.4	62.5 ± 0.0
	Aminoglycosides	88.5 ± 11.8	63.0 ± 23.1	96.5 ± 6.1	100.0 ± 0.0
	Coumarin glycosides	40.6 ± 0.0	55.6 ± 0.0	73.7 ± 0.0	50.0 ± 0.0
	Chloramphenicol	31.3 ± 0.0	33.3 ± 0.0	68.4 ± 0.0	62.5 ± 0.0
	Nitrofurantoin	81.3 ± 0.0	11.1 ± 0.0	89.5 ± 0.0	62.5 ± 0.0
	Rifampin	15.6 ± 0.0	55.6 ± 0.0	47.4 ± 0.0	25.0 ± 0.0
	Sulfonamide compounds S3	75.0 ± 0.0	66.7 ± 0.0	84.2 ± 0.0	75.0 ± 0.0
	Tetracyclines	28.1 ± 0.0	0.0 ± 0.0	31.6 ± 0.0	0.0 ± 0.0
	Trimethoprim/sulfonamides	53.1 ± 0.0	44.4 ± 0.0	94.7 ± 0.0	50.0 ± 0.0
Narrow spectrum (G +)	Macrolides	43.8 ± 13.3	16.7 ± 7.9	86.8 ± 3.7	87.5 ± 0.0
	Lincosamides	75.0 ± 17.7	83.3 ± 7.9	94.7 ± 0.0	93.8 ± 8.8
	Fatty acyls	93.8 ± 0.0	77.8 ± 0.0	100.0 ± 0.0	87.5 ± 0.0
	Glycopeptides	28.1 ± 0.0	44.4 ± 0.0	73.7 ± 0.0	100.0 ± 0.0
Anaerobes	Metronidazole	93.8 ± 0.0	77.8 ± 0.0	94.7 ± 0.0	100.0 ± 0.0

Percentage of resistant strains to the 17 tested antibiotic classes was indicated for different bacterial phyla and Gram-stain type (average and standard deviation values).

The AR profile of the cave isolates indicated the presence of multi-drug (MDR) (25), extended-drug (XDR) (8), and pan-drug (PDR) (2) resistance phenotypes, according to the definitions proposed by Magiorakos et al.^51^, described below in “Methods” section. Among these, *Arthrobacter psychrolactophilus* SC7AB.1 isolated from the 400-years old ice layer was resistant only to nalidixic acid and cefixime (Supplementary Table S3). At the opposite pole, the SC97A.1 and SC97A.2 *Pseudomonas* strains isolated from the 13,000-years old ice layer showed a PDR phenotype to all antibiotics.

**Figure 2:**
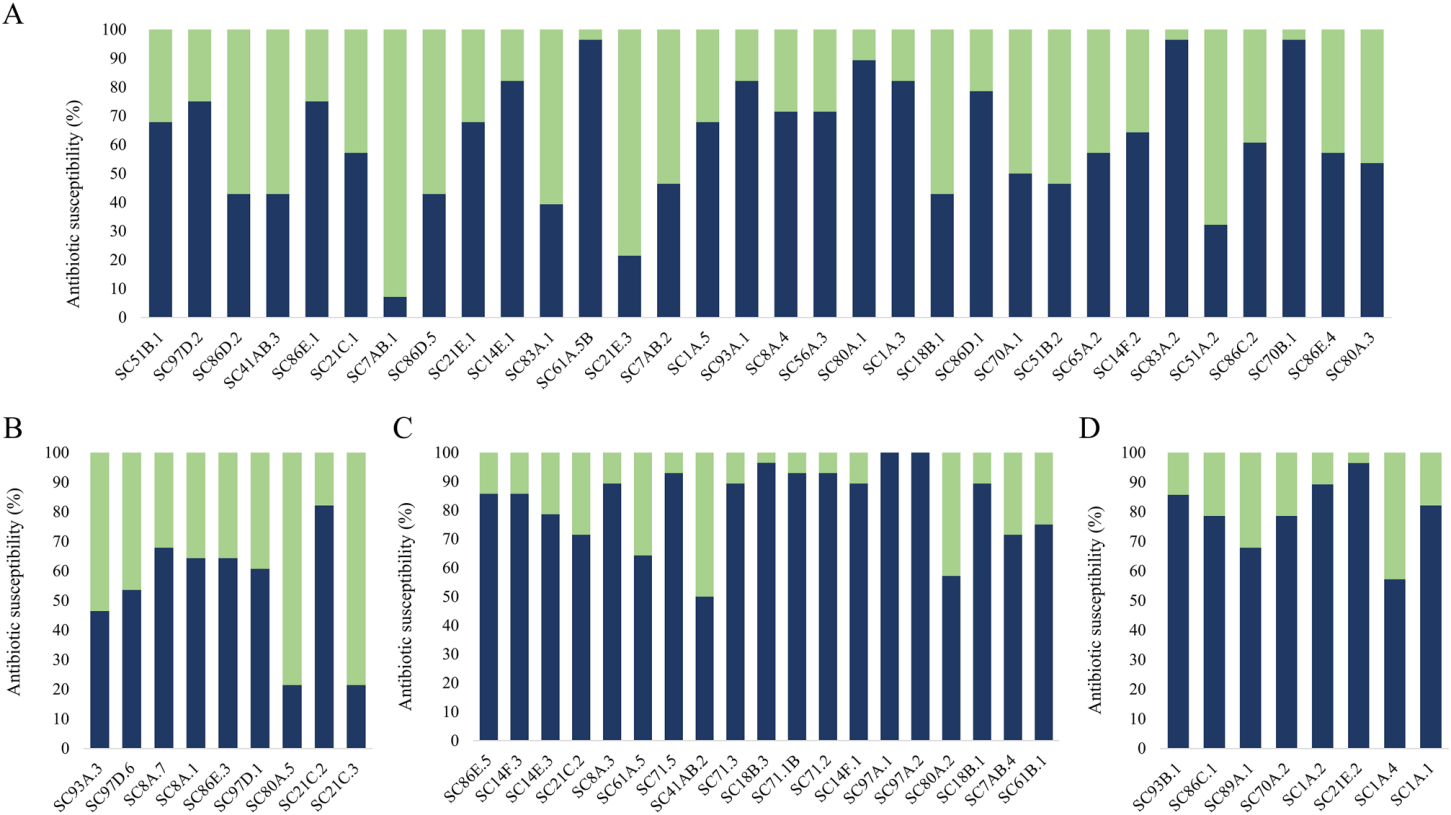
Antimicrobial susceptibility of cave ice bacterial isolates. The percentage of susceptible under standard dose (green), and resistant (blue) strains to the 28 tested antibiotics was indicated for the strains belonging to phyla Actinobacteria (**A**), Firmicutes (**B**), Proteobacteria (**C**), and Bacteroidetes (**D**).

Relative to the total number of drugs tested (Fig. 2), the antibiotic susceptibility of cave bacteria belonging to different phyla showed large taxonomic-related variations. Gram-positive isolates displayed the highest resistance to metronidazole, fatty acyls, lincosamides, cephalosporins and aminoglycosides, and to nitrofurantoin and nalidixic acid in the case of Actinobacteria (Table 1). Among these, *Microbacterium pygmaeum* SC83A.2, *Pseudarthrobacter oxydans* SC70B.1 and *Dietzia* sp. SC61A.5B displayed a XDR phenotype, while *G. tibetensis* SC21E.3, *Mycetocola manganoxydans* SC51A.2 and *A. psychrolactophilus* SC7AB.1 were susceptible to most of the tested antibiotics (Fig. 2A). The highest AR of Firmicutes was determined for *Sporosarcina globispora* SC21C.2 (MDR) and *Bacillus* sp. SC8A.7 (19 antibiotics) (Fig. 2B, Supplementary Table S3), while *Sporosarcina* sp. SC21C.3, *Bacillus safensis* SC93A.3 and *P. macmurdoensis* SC80A.5 were resistant to only 6, 13 and 6 antibiotics, respectively (Fig. 2B).

More than 50% of the Gram-negative cave ice bacteria were resistant to all tested antibiotic classes, excepting rifampin, tetracyclines and carbapenems in the case of Proteobacteria (Table 1). The majority of Proteobacteria (16 isolates) showed a MDR phenotype, while *E. caeni* SC41AB.2, *Caulobacter* sp. SC61A.5 and *P. glaciei* SC80A.2 were only resistant to 14, 18 and 16 antibiotics, respectively (Fig. 2C). Noticeably, *Pseudomonas* spp. SC97A.1 and SC97A.2 strains presented a PDR profile (Fig. 2C). Gram-negative Bacteroidetes strains were also characterized by a high AR with all 8 strains resistant to > 50% of the tested antibiotics (Fig. 2D). Among these, *M. phyllosphaerae* SC21E.2 showed the broadest (27 antibiotics) and *P. bambusae* SC1A.4 the most limited AR (16 antibiotics).

The highest resistance rate to broad-spectrum aminoglycosides was displayed by Bacteroidetes (100%), Proteobacteria (96.5%) and Actinobacteria (88.5%) strains, while only 62.9% of Firmicutes were resistant to this class (Table 1). A similar resistance rate against cephalosporins was observed for Proteobacteria and Bacteroidetes isolates (92.1%, 95.8%), unlike Actinobacteria and Firmicutes (76%, 79.6%) (Table 1). All Firmicutes and Bacteroidetes strains were susceptible to tetracyclines, while 28.1% Actinobacteria and 31.58% Proteobacteria were resistant to this antibiotic. Firmicutes strains were completely susceptible to carbapenems, unlike Actinobacteria (9.4%) and Proteobacteria (31.6%) isolates (Table 1).

Among the retrieved cave isolates, Actinobacteria showed a variable resistance profile, 93.8% being resistant to metronidazole and only 9.4% to carbapenems. Firmicutes were highly resistant to lincosamides (83.3%) while only 11.1% to nitrofurantoin, and completely susceptible to carbapenems and tetracyclines (Table 1). Gram-negative Proteobacteria strains had a variable resistance profile, ranging from 31.6% for carbapenems and tetracyclines, to 100% for fatty acyls, and for Bacteroidetes from 25% (rifampin) to 100% (aminoglycosides, glycopeptides and metronidazole), with total susceptibility to tetracyclines (Table 1).

### Functional variability of ice core isolates

Based on the antimicrobial susceptibility profile (Supplementary Table S3) and age of the ice substrate (Supplementary Table S2), 11 cave isolates were selected along the ice core chronosequence for further characterization comprising five Gram-positive Actinobacteria (*A. psychrolactophilus* SC7AB.1, *Dietzia* sp. SC61A.5B, *M. ginsengiterrae* SC1A.5, *M. pygmaeum* SC65A.2 and *Pseudarthrobacter* sp. SC86E.4), five Gram-negative Proteobacteria (*Candidimonas bauzanensis* SC8A.3, *Caulobacter* sp. SC61A.5, *Delftia* sp. SC71.5, *P. brenneri* SC97A.1 and *P. grimontii* SC97A.2), and the Firmicutes *B. toyonensis* SC86E.3 strain (Supplementary Table S1).

Screening of enzymatic activities using API ZYM test system^52^ showed a distinct enzymatic profile of the tested cave bacteria (Table 2). Although none of the isolates hydrolyzed all 19 substrates, *A. psychrolactophilus* SC7AB.1, *M. ginsengiterrae* SC1A.5 and *M. pygmaeum* SC65A.2 tested positive for 74% of activities. According to evaluation codes, high leucine arylamidase and naphthol-AS-BI-phosphohydrolase activities were identified for all isolates, while no α-chymotrypsin and α-fucosidase activities were detected (Table 2). The only strain showing a weak N-acetyl-β-glucosaminidase activity was *M. pygmaeum* SC65A.2. *P. brenneri* SC97A.1, *A. psychrolactophilus* SC7AB.1, *C. bauzanensis* SC8A.3, *Delftia* sp. SC71.5 and *Dietzia* sp. SC61A.5B had a high alkaline phosphatase activity, unlike *B. toyonensis* SC86E.3 and *M. pygmaeum* SC65A.2 showing no activity. Hydrolysis of 2-naphthyl-αd-glucopyranoside was prominent for *A. psychrolactophilus* SC7AB.1, *B. toyonensis* SC86E.3, *Caulobacter* sp. SC61A.5, *Dietzia* sp. SC61A.5B, *M. ginsengiterrae* SC1A.5 and *M. pygmaeum* SC65A.2. The lowest number of hydrolyzed substrates (5 out of 19) was recorded for *Delftia* sp. SC71.5.

**Table 2:**
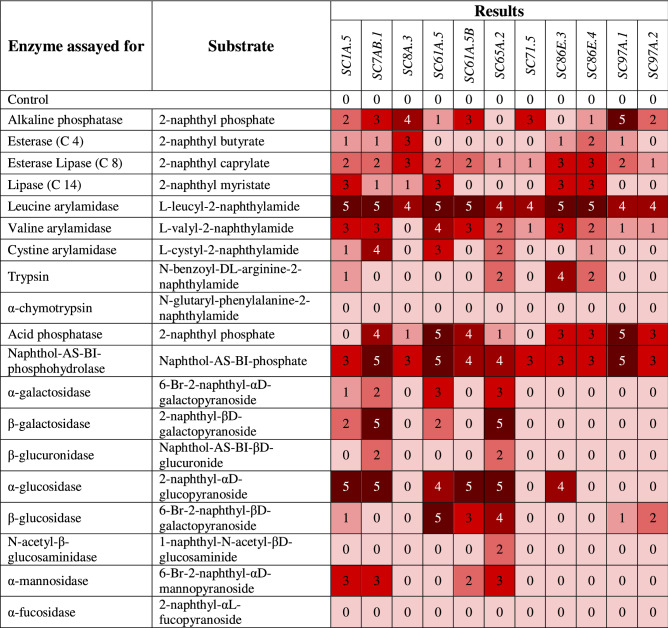
API ZYM enzymatic activity of cave ice isolated strains.

The color intensity decreased with the level of enzymatic activity from 5 (high) to 0 (no activity). The numbers correspond to the amount of hydrolyzed substrate (nmol), as indicated in “Methods” section.

Complementary API 20NE tests revealed a distinct substrate utilization profile of the selected cave strains (Table 3). All 11 isolates could hydrolyze β-glucosidase (esculin), while none of the strains were able to reduce nitrates and tested negative for indole production, glucose fermentation and arginine dihydrolysis. Nitrate reduction was observed for most of the strains except for *Caulobacter* sp. SC61A.5, *M. ginsengiterrae* SC1A.5 and *P. brenneri* SC97A.1. Only four isolates were able to metabolize urea. Gelatin hydrolysis was limited to *B. toyonensis* SC86E.3 and *M. ginsengiterrae* SC1A.5. *A. psychrolactophilus* SC7AB.1, *M. ginsengiterrae* SC1A.5, *M. pygmaeum* SC65A.2 and *Pseudarthrobacter* sp. SC86E. Four cave bacteria tested positive for β-galactosidase, and cytochrome oxidase activity was measured for five strains (Table 3).

**Table 3:** API 20NE enzymatic assay.

Test	Reaction/enzyme	Active ingredient	Results										
			SC1A.5	SC7AB.1	SC8A.3	SC61A.5	SC61A.5B	SC65A.2	SC71.5	SC86E.3	SC86E.4	SC97A.1	SC97A.2
NO3	Reduction of nitrates to nitrites	Potassium nitrate	−	+	+	−	+	+	+	+	+	−	+
	Reduction of nitrates to nitrogen		−	−	−	−	−	−	−	−	−	−	−
TRP	Indole production	l-tryptophane	−	−	−	−	−	−	−	−	−	−	−
GLU	fermentation (glucose)	d-glucose	−	−	−	−	−	−	−	−	−	−	−
ADH	Arginine dihydrolase	l-arginine	−	−	−	−	−	−	−	−	−	−	−
URE	Urease	Urea	−	−	−	+	+	+	−	−	+	−	−
ESC	Hydrolysis (β-glucosidase)	Esculin ferric citrate	+	+	+	+	+	+	+	+	+	+	+
GEL	Hydrolysis (protease)	Gelatin (bovine origin)	+	+	−	+	−	−	−	+	−	+	−
PNPG	β-Galactosidase	4-Nitrophenyl-βd-galactopyranoside	+	+	−	−	−	+	−	−	+	−	−
GLU	Assimilation (glucose)		+	+	+	+	+	+	−	+	+	+	+
ARA	Assimilation (arabinose)		+	+	+	+	+	+	−	−	−	+	+
MNE	Assimilation (mannose)		+	+	+	+	+	+	−	−	+	+	+
MAN	Assimilation (mannitol)		+	+	+	+	+	+	−	−	−	+	+
NAG	Assimilation (*N*-acetyl-glucosamine)		−	+	−	−	+	+	−	+	−	+	+
MAL	Assimilation (maltose)		+	+	+	+	−	+	−	+	+	−	−
GNT	Assimilation (potassium gluconate)		+	+	+	−	+	+	+	−	+	+	+
CAP	Assimilation (capric acid)		−	−	+	−	+	−	+	−	−	+	+
ADI	Assimilation (adipic acid)		−	−	+	−	−	−	+	−	−	+	−
MLT	Assimilation (malate)		+	+	+	+	+	+	+	+	+	+	+
CIT	Assimilation (trisodium citrate)		−	+	+	+	+	+	−	+	+	+	+
PAC	Assimilation (phenylacetic acid)		−	−	−	−	−	+	−	−	+	+	−
OX	Cytochrome oxidase		−	−	+	−	+	−	+	−	−	+	+

In addition, a variable substrate assimilation pattern was observed (Table 3). All strains were able to use malate, and 10 of the isolates could metabolize glucose. *P. brenneri* SC97A.1 used all tested substrates except for maltose, followed by *C. bauzanensis* SC8A.3 and *M. pygmaeum* SC65A.2 (10 substrates). Meanwhile, a reduced assimilation capacity was observed for *Delftia* sp. SC71.5 and *B. toyonensis* SC86E.3 testing positive for 4 and 5 substrates, respectively (Table 3).

### Antimicrobial activity of isolates from Scarisoara cave ice core

Eleven cave isolates showing optimal growth parameters and diverse resistance profiles have been selected for their antimicrobial activity against 2 ATCC strains and 20 clinical pathogenic bacteria, exhibiting epidemiologically important resistance phenotypes (Supplementary Table S4).

All selected cave isolates showed antimicrobial activity against *Staphylococcus aureus* ATCC 25923, *P. aeruginosa* CN11 and MRSA 19081 F1 (Table 4). Ten cave strains inhibited *Enterobacter cloacae* 19069 ONE2, *E. cloacae* 19069 ONE3, and *P. aeruginosa* 19053 CNE5, while nine isolates had activity against *Escherichia coli* ATCC 25922, with a low inhibition of clinical *Klebsiella* 19094 strains CK1, KC2, and CK3, and of three *Enterococcus faecium* strains (Table 4).

**Table 4:** Antimicrobial activity of Scarisoara ice cave bacteria.

Test pathogen	Cave isolates										
	SC1A.5	SC7AB.1	SC8A.3	SC61A.5	SC61A.5B	SC65A.2	SC71.5	SC86E.3	SC86E.4	SC97A.1	SC97A.2
*Staphylococcus aureus* ATCC 25923	+	+	+	+	+	+	+	+	+	+	+
*Escherichia coli* ATCC 25922	+	+	+	+	+	+	+	−	+	−	+
*Enterobacter asburiae* 19069 ONE1	−	−	−	−	−	−	−	−	−	−	−
*Enterobacter cloacae* 19069 ONE2	+	+	−	+	+	+	+	+	+	+	+
*Enterobacter cloacae* 19069 ONE3	+	+	+	+	+	+	+	+	−	+	+
*Pseudomonas* CN11	+	+	+	+	+	+	+	+	+	+	+
*Pseudomonas aeruginosa* 19053 CNE5	−	+	+	+	+	+	+	+	+	+	+
*Pseudomonas aeruginosa* 19053 CNE6	−	−	+	+	+	+	−	−	−	+	+
MRSA 19081 F1	+	+	+	+	+	+	+	+	+	+	+
MRSA 19081 S1	−	−	−	−	−	−	−	−	−	−	−
MRSA 388	−	−	−	−	−	−	−	−	−	−	−
*Klebsiella* 8	−	−	−	−	−	−	−	−	−	−	−
*Klebsiella* 19094 CK1	−	−	−	−	−	−	+	−	−	−	+
*Klebsiella* 19094 CK2	−	−	−	−	+	+	−	−	−	−	+
*Klebsiella* 19094 CK3	−	−	−	−	−	−	−	−	−	−	+
*Acinetobacter* 19047 ENE4	−	−	−	−	−	−	−	−	−	−	−
*Acinetobacter* 19047 CNE5	−	−	−	−	−	−	−	−	−	−	−
*Acinetobacter* 19047 CNE3	−	−	−	−	−	−	−	−	−	−	−
*Acinetobacter* 18032 C3	−	−	−	−	−	−	−	−	−	−	−
*Enterococcus falcium* 19040 E1	+	−	−	−	−	+	−	−	−	−	+
*Enterococcus falcium* 19040 E2	+	−	−	−	−	+	−	−	−	−	+
*Enterococcus falcium* 19040 E3	+	−	−	−	+	−	−	−	−	−	+
Total	9	7	7	8	10	11	8	6	6	7	14

The inhibitory response of the ATCC and clinical isolates corresponded to (+) presence of inhibition ring, and (−) absence of inhibition ring, as indicated in “Methods” section.

A broad antimicrobial spectrum was determined for the 13,000 cal BP *P. grimontii* SC97A.2 strain, active against most tested pathogens (Table 4). Interestingly, the methicillin susceptible and resistant *S. aureus* strains, and the *P. aeruginosa* pathogens were inhibited by all 11 cave isolates. *M. pygmaeum* SC65A.2 and *Dietzia* sp. SC61A.5B isolated from 5000 cal BP Scarisoara ice inhibited 11 and 10 of the tested pathogens, respectively, and *M. ginsengiterrae* SC1A.5 (100 cal BP) showed activity against 9 pathogens. No antimicrobial effect was recorded against one *Enterobacter*, two methicillin resistant *S. aureus*, one *Klebsiella,* and all *Acinetobacter* sp. strains (Table 4).

## Discussion

This study represents the first characterization of 68 distinct cold-active strains isolated from a 13,000-years old underground ice core of Scarisoara ice cave (Romania) focused on their growth temperature, antibiotic resistance, enzymatic and antimicrobial activity profiles.

### Cold-adapted bacterial isolates from 13,000-years old cave ice

Despite the lower microbial density found in Scarisoara ice strata^18,22^ as compared to glacier ice^53^, the cave isolates exhibited a large diversity, covering the main phyla commonly found in frozen habitats, i.e. Actinobacteria, Proteobacteria, Firmicutes and Bacteroidetes^1,7,43^. Among these, an important fraction (17 strains) showed a low 16S rRNA gene identity with previously reported bacteria (Supplementary Table S1), suggesting the existence of possible new species in this habitat. Also, the *Aeromicrobium* sp. and *Chryseobacterium* sp. strains isolated from the ice strata accumulated during the Late Glacial Period (LGP, > 12,000-years BP) appeared unrelated to other representatives of these genera. Interestingly, all of these LGP-originating bacteria were psychrotrophs able to grow above 30 °C.

From Actinobacteria, a prevalent microbial taxa in cold environments^7,54,55^, the *Cryobacterium* spp*.* Scarisoara isolates are, to our knowledge, the first retrieved from ice accumulated in caves^53,56–60^ and *Salinibacterium xinjiangens*^61–63^ was cultivated for the first time from ice deposits. Amongst Proteobacteria, *Pseudomonas* (13,000 years old ice) and *Psychrobacter glaciei* (8000 years old ice) strains were isolated for the first time from the ice core of this cave, adding this habitat to other frozen environments colonized by these bacteria^64–67^. Interestingly, the *Phyllobacterium* cave isolates from 700 and 7000-years old ice homologous to plants-associated species^68,69^ could originate from the abundant vegetation above the cave^70^. Firmicutes strains belonging to *Bacillus* and *Sporosarcina* genera were retrieved from different Scarisoara ice strata, confirming the resilience of these genera in recent and old icy habitats^8,9,53,54,71–75^. The presence of *Flavobacterium glaciei* in 100-years old Scarisoara ice, previously reported in China No.1 glacier^76^, highlighted a wide geographical distribution of this psychrophilic species. In addition, the recovery from 100-years old ice of *Pedobacter* strains closely related to oil and aquatic strains^77,78^ confirmed their adaptation to frozen habitats^9,79^. Meanwhile, the scarce recovery of Bacteroidetes strains from Scarisoara ice suggested the high sensitivity of this phylum^67^ to environmental variations.

### Antibiotic resistance in Late Glacial cave ice core

Scarisoara ice cave isolates showed high resistance to different antibiotic classes including the ones drastically affected by the emergence of resistant strains in the clinical sector. Most Scarisoara isolates exhibiting MDR and XDR phenotypes thrived in ice strata formed at least 1000-years ago. Bacterial strains with the broadest (PDR) resistance belonged to *Pseudomonas* species originating from 13,000-years old ice, similar to Arctic^9^ and Antarctic^80^ homologs.

Most of the cave isolates with broad AR were Gram-negative belonging to Proteobacteria (6) and Bacteroidetes (1), while *Arthrobacter psychrolactophilus* SC7AB.1 isolated from 400-years old ice was resistant only to nalidixic acid and cefixime, (Supplementary Table S3). Although not specific for cold-habitats^81,82^, a high tetracycline susceptibility rate of the cave isolates was observed, also reported in Arctic bacteria^9^.

A different response to antibiotics of the cave isolates was observed for the 5000-years old ice *Dietzia* sp. SC61A.5B (XDR) as compared to their homologs *D. maris*^83^ and *D. papillomatosis*^84^ susceptible to most antibiotics. The two XDR *Paralcaligenes* cave strains SC18B.3 (900-years BP) and SC71.3 (7000-years BP) appeared to have an opposite phenotype as their homologs isolated from soil resistant to only lincomycin, oleandomycin, rifampicin, and vancomycin^85^. Other four XDR cave isolates belonging to *Flavobacterium, Paralcaligenes, Phenylobacterium* and *Microbacterium* genera isolated from up to 9000-years old ice (Supplementary Table S1) revealed a broader AR than the corresponding species from clinical isolates^86^, but also Antarctic^80^ and Arctic^9,54^ cold habitats.

Similar susceptibility patterns were observed for *Brevundimonas* SC86E.5 isolated from 10,000-years old Scarisoara ice and Antarctic lake homologous species^80^. Some cave isolates showed a species specific antibiotic resistance profile independent on the age and type of habitat, as in the case of the MDR *P. trifolii* SC14F.1 (700-years BP) and XDR *P. loti* SC71.2 (7000-years BP) susceptible to different antibiotics (Supplementary Table S3), although with similar response as their best match strains originating from plants^68,69^. Comparable AR profile was also observed for *Mucilaginibacter* species from frozen environments^87,88^ and Scarisoara SC21E.2 strain (XDR). Interestingly, *Delftia* sp. SC71.5 from 7000-years BP cave ice shared a similar resistance profile with clinical homologs^89^, although the low 16S rRNA gene identity (95%) could indicate a more distal cave strain ancestor.

The prevalence of MDR found in the 68 isolated strains revealed the existence of highly diverse resistome in old samples (5000–30,000-years old) from permafrost, Arctic soil, and a deeper cave site disconnected from the surface for over 4 Myr^33,81^, suggesting a correlation between AR and cold-adaptation mechanisms via horizontal gene transfer (HGT)^90^. In our study, the high incidence of XDR and PDR phenotypes recovered from layers of old ice sealed between 1000–13,000 years could be associated with the high relative abundance of phyla Actinobacteria and Proteobateria^18^. Actinobacteria are known for their ability to produce bioactive compounds and for their multiple resistance to antibiotics^91^, and Proteobacteria constitute a recognized group susceptible to acquire AR genes by HGT^35,92,93^. In some cases, MDR could be conferred by cross-resistance as observed for cave isolates resistant to tetracycline, various β-lactams, all aminoglycosides and almost all macrolides. A similar pattern of cross-resistance was observed in methicillin-resistant *S. aureus* strains^94^.

### Climate impact on the AR reservoir of cave ice bacteria

Climate variation during ice deposition appeared to model the composition of bacterial^18,22,27,28^ and fungal^24,29^ communities entrapped in perennial ice accumulated in Scarisoara ice cave, in particular during the cold and dry Little Ice Age (LIA, AD 1250–AD 1860), and the warm and wet intervals Medieval Warm Period (MWP) (AD 800–AD 1250) and Mid-Holocene Warm Period (MHWP; 5500–6500 years BP)^16,17,95^. In this respect, uncultured prokaryotic communities from LIA and MWP formed ice strata of this cave appeared to be dominated by Firmicutes or Actinobacteria^28^, while Proteobacteria dominated both the total and potentially active bacterial communities contained in MHWP ice^18^ accumulated during to Sahara Desert formation period^96^.

**Figure 3:**
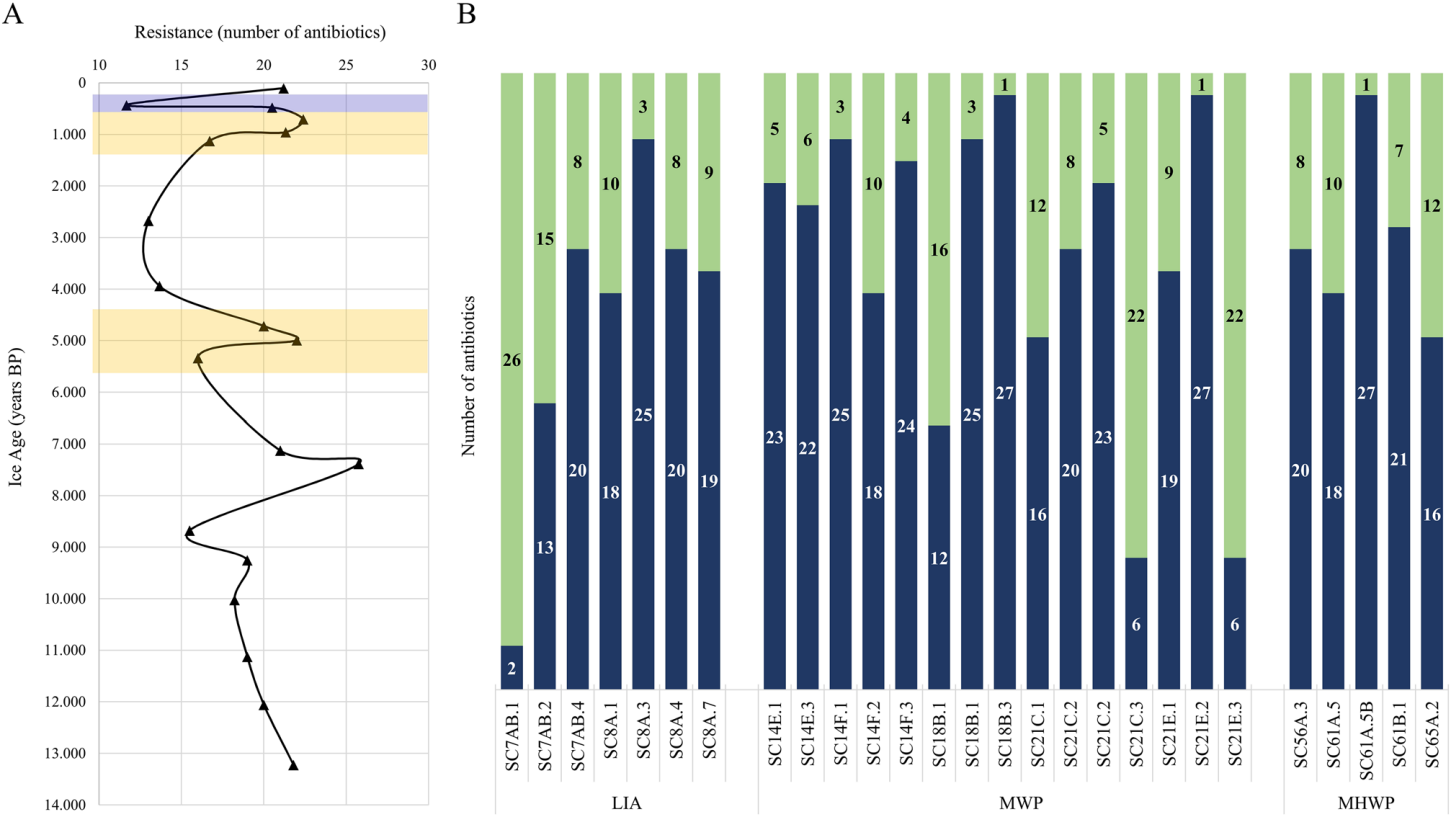
Impact of substrate age and climate during ice deposition on the antimicrobial susceptibility of bacterial isolates from Scarisoara Ice Cave. (**A**) Resistance profile of the cave ice chronosequence. (**B**) Antibiotic resistance (blue) and susceptibility at standard dose (green) to 28 antibiotics (Supplementary Table S3) of cave isolates from ice layers accumulated during Little Ice Age (LIA; 430–475 years BP), Medieval Warm Period (MWP; 953–1124 years BP) and Mid Holocene Warm Period (MHWP; 4751–5335 years BP) (Supplementary Table S2).

The current data (Fig. 3A) revealed a variable AR profile of cave isolates along the 13,000-years BP ice core, with broader resistance (MDR and PDR phenotypes) in recently formed ice, the warmer periods MWP, MHWP, and 7000-years old ice, alternating with higher susceptibility during LIA, 3000–4000-years BP, 5500-years BP, and 9000-years BP periods. An increased resistance was also observed for isolates from older ice extended to Late Glacial period (12,000–13,000-years BP) (Fig. 3A).

Bacterial isolates from cave ice deposits accumulated during LIA, MWP and MHWP intervals (Fig. 3B) showed a variable AR. Among LIA isolates, a broad resistance (13–25 antibiotics) was observed for *C. bauzanensis* SC8A.3, *R. argentea* SC7AB.4 and *M. hydrocarbonoxydans* SC8A.4, *Bacillus* spp. SC8A.7, SC8A.1 and *L. antarctica* SC7AB.2, while *A. psychrolactophilus* SC7AB.1 (Actinobacteria) was highly sensitive to most of the antibiotics (Fig. 3B).

Most of the isolates from MWP and MHWP ice had broad AR, including 11 MDR strains mostly belonging to Proteobacteria and the XDR *Dietzia* sp. SC61A.5B (Fig. 3B). For these warm and wet climate intervals, the most susceptible bacterial strains belonged to Actinobacteria (*G. tibetensis* SC21E.3, *M. hydrocarbonoxydans* SC18B.1, *A. psychrochitiniphilus* SC21C.1 and *M. pygmaeum* SC65A.2 and SC14F.2), and Firmicutes (*Sporosarcina* sp. SC21C.3) species (Fig. 3B). Therefore, the broad resistance of resilient Proteobacteria representatives from cave ice could be a hint in searching new candidates for untangling environmental AR mechanisms.

### Applicative potential of Scarisoara cave ice bacterial isolates

To overcome the MDR in microorganisms with medical and industrial relevance, screening of microbiomes from unexplored old habitats is crucial for understanding their evolution and discovery of new antibiotics. Microorganisms from cold environments, mostly belonging to Actinobacteria, are able to inhibit opportunistic human pathogens, constituting largely unexplored reservoirs of new natural antimicrobials^49,97–99^. Cold-adapted bacteria were also reported as a source of enzymes with enhanced stability and novel characteristics^23,100^.

The corroborated characterization of the 11 bacterial isolates from Scarisoara ice cave revealed new psychrotolerant strains showing high AR and antimicrobial activity against at least one or more clinical Gram-negative and Gram-positive pathogens (Table 4), in addition to a series of enzymatic activities as valuable catalysts candidates for low temperature industrial processes. To our knowledge, these data on microbial isolates from the 13,000-years old underground ice deposits of Scarisoara cave provided the first glimpse on the ice caves’ potential as reservoirs of new antimicrobial biomolecules.

Among cold habitats bacteria, *Pseudomonas* species presented a broad antimicrobial activity^43,49^. In the case of Scarisoara isolates, the two *Pseudomonas* strains from 13,000 cal BP ice strata (*P. brenneri* SC97A.1 and *P. grimontii* SC97A) showed a high antimicrobial activity against 7 and 14 pathogens, respectively, the later one with extensive preference for Gram-negative clinical isolates. Considering their PDR phenotype, these strains could constitute important candidates for studying the resistome of millennia-old bacteria and AR evolution. Moreover, their positive testing for acid phosphatase and leucine arylamidase activities with applications in food industry^101^ and alkaline phosphatase, commonly used for clinical diagnostics and dairy industry^102^ constitute promising leads for new biocatalysts.

The 5000-years old *Dietzia sp*. SC61A.5B showing a XDR phenotype also inhibited the growth of Gram-negative pathogens. Similar to *Pseudomonas* cave isolates, this Scarisoara strain showed high leucine/valine arylamidase and acid phosphatase activities, important catalysts for food processing^101^. Moreover, *M. pygmaeum* SC83A.2 isolated from 9000-years BP cave ice displayed a high antimicrobial activity mainly against Gram negative pathogens, associated with XDR phenotype. The broader AR of this strain as compared to Artic isolates^9^ uncovered a valuable bacterial candidate for exploring new antimicrobial mechanisms.

*Caulobacter* sp. SC61A.5 cave isolate (5000-years old ice) was also one of the strains with broader antimicrobial activity against Gram-negative pathogens and broad AR phenotype, unlike other representatives of this genus^41^. This psychrotrophic ice bacterium also presented α- and β-glucosidase activities with putative extensive applications^103^. Similar to *Pseudomonas* and *Dietzia* cave isolates, a high Leu/Val arylamidase and acid phosphatase activity was measured for this strain.

*Arthrobacter psychrolactophilus* SC7AB.1 showed a particular AR to fluoroquinolone and cephalosporins, and antimicrobial activity against Gram-negative and Gram-positive pathogens, in addition to a variety of enzymatic activities as putative source of cold-active catalysts. The XDR phenotype of *Delftia* sp. SC71.5 isolated from 7000-old cave ice, unlike the homologous soil strain susceptible to broad spectrum antibiotics^104,105^, and the broad antimicrobial activity against Gram-negative pathogens recommend theses strains as novel candidates for studying the environmental resistome and for isolation of putative active biomolecules.

Most of the cave isolates tested positive for acid phosphatase activity used in food industry, constituting valuable catalyst candidates for low temperature food processing^101^. Cold-active lipase activity for a wide range of applications in biofuels and pharmaceutical industry^106^, detergents^107^, and food processing^108^ was also identified in most of the cave ice isolates.

## Conclusion

In addition to our previous reports on cultivable bacteria from ice layers formed during the last 900-years in Scarisoara ice cave^21,22^, the current study adds to the knowledge on functional characteristics of isolated bacteria from this underground perennial ice core. The isolated bacterial strains belonging to the four major phyla ubiquitous for frozen environments confirmed their viability along the 13,000 cal BP cave ice, and revealed their extended antibiotic resistance profiles, as a pioneering survey for this type of habitat for understanding the environmental resistome evolution.

Although with a lower microbial density as compared to glacier ice, our cave isolates belonging to Actinobacteria, Proteobacteria, Firmicutes and Bacteroidetes exhibited a large diversity encompassing putative new representatives for this habitat or worldwide. Many of these strains preserved in millennia-old ice from a secluded type of icy habitat exhibited a large spectrum of antimicrobial resistance, highlighting their important contribution to the environmental resistome, and the potential risk of releasing antibiotic resistant bacteria in the water and soil due to temperature increase and ice melting. However, further studies are needed to identify the associated AR genes, their localization and transferability potential. In addition, the psychrotrophic isolates highlighted significant antimicrobial and catalytic activities, being thus valuable candidates for biomedical and biotechnological applications. Overall, due to their extended AR profile as compared to that of the reported homologs and the antimicrobial activity of all isolates, in particular against the Gram-negative pathogens raising global treatment limitations, these cave bacterial strains retrieved from up to 13,000-years old ice could provide important clues for understanding the evolution of natural and clinical resistance and for developing novel antimicrobial strategies.

## Methods

### Ice samples

Ice sampling was performed by vertical drilling to a depth of 25.33 m into the perennial ice block of Scarisoara Ice Cave resulting in 97 ice core fragments covering a chronology of up to 13,000 calibrated years before present (cal BP) ice, as previously described^18^. All ice samples were collected under aseptic conditions by flaming the drilling equipment and using sterile 1-L plastic bags, transported to the laboratory under permanent frozen conditions where they were stored at − 20 °C until processed^18^.

### Isolation of bacterial strains

A series of 28 core ice samples covering an age span of 92 ± 26 cal BP to 13,098 ± 29 cal BP at 100–300 years old interval^18^ were thawed at 4 °C in the absence of light, and inoculated in 10 mL R2B liquid media (Reasoner’s 2B broth, Melford Biolaboratories Ltd., UK). The enriched samples were incubated at 4 °C and 15 °C in shaker incubators (160 rpm) for up to 120 days. The enriched cultures (100 µL) were used to inoculate R2A Agar (Reasoner’s 2A agar, Merck Millipore, Germany) plates using a 10^0^–10^–10^ serial dilution, and incubated at 4 °C and 15 °C for 2 to 4 weeks. Morphologically different colonies were isolated and purified under the same growth conditions.

### Isolates identification

Bacterial isolates were identified by 16S rRNA gene sequencing. Total DNA was extracted using DNeasy Blood & Tissue Kit (Qiagen, USA) and the 16S rRNA gene fragments were amplified by PCR as previously described^21^. After purification (Invitrogen, USA), the amplicons were sequenced using the amplification primers (Macrogen, Netherlands).

Chimera analysis of the sequences and low-quality aligned regions were eliminated using CodonCode Aligner version 8.0.1 (CodonCode Corporation, www.codoncode.com), and the closest phylotype was assigned by BLAST alignment^109^.

### Bacterial strains characterization

#### Growth temperature

The minimum and maximum growth temperatures of the isolated strains were determined by cultivation at 4 °C, 10 °C, 15 °C, 20 °C, 25 °C, 30 °C and 37 °C up to 14 days on Tryptic Soy Broth-agar (TSA; Scharlab, Spain) solid medium.

#### Antibiotic susceptibility test

The antimicrobial susceptibility analysis was carried out using the disk diffusion method^110^. The isolates were cultivated on R2A agar and TSA agar plates at 15 °C for 2–5 days. The antibiotic susceptibility profile was determined after incubation at 37 °C for 24 h, using *Stahylococcus aureus* ATCC 25923 (Thermo Scientific, USA) as control strain^110^. Based on the number of antibiotics with inhibitory effect, the strains were assigned as MDR, defined as non-susceptible to at least one agent in three or more antimicrobial categories, XDR as non-susceptible to at least one agent in all but two or fewer antimicrobial categories, and PDR, defined as non-susceptible to all agents in all antimicrobial categories. According to these definitions, our strains exhibiting a MDR phenotype were resistant to 20–25 antibiotics, from at least three different classes, the XDR ones were resistant to 26–27 antibiotics, and those exhibiting a PDR were resistant to all tested 28 antibiotics^51^.

#### Biochemical characterization

Functional characterization of selected bacterial isolates was performed using API ZYM and API 20NE strips (BioMérieux, France) according to the manufacturer protocol, with incubation at 15 °C. The enzymatic activity was evaluated based on the number of nmol of hydrolyzed substrate as (0): no activity, (1 and 2): low activity (5 nmol and 10 nmol, respectively), (3): moderate activity (20 nmol), (4 and 5): high activity (30 nmol and ≥ 40 nmol, respectively)^111^.

#### Antimicrobial activity

The antimicrobial activity of the ice cave isolates was tested against two reference strains, i.e. *Staphylococcus aureus* ATCC 25923 and *Escherichia coli* ATCC 25922 (Thermo Scientific, USA), and 20 clinical isolates from the Research Institute of The University of Bucharest Microbial Collection (Supplementary Table S4), using the Kirby–Bauer method^112^. The cave isolates were cultivated in 20 mL Tryptic Soy Broth (TBS) medium (Merck Millipore, Germany) at 15 °C, under agitation (160 rpm), for up to 14 days, and the cells were harvested by centrifugation at 7500 rpm for 20 min. The supernatant was filtered-sterilized using 0.22 µm syringe filters (Merck Millipore, Germany), and concentrated to 1 mL with a vacuum Concentrator Plus Complete system (Eppendorf, USA). The pathogen (test) strains were inoculated on Mueller–Hinton agar (MHA) solid medium (2 g L^−1^ beef extract, 17.5 g L^−1^ casein acid hydrolysate, 1.5 g L^−1^ starch, and 17 g L^−1^ agar) and grown at 37 °C for 18 h. One colony from each test strain was resuspended in physiological saline solution to 0.5 McFarland turbidity, and further plated on MHA solid medium covering uniformly the plate surface. Immediately after pathogen plating, 5 µL samples of concentrated extracts of cave bacteria were added as single drops in separated quadrants (6 per plate). After incubation at 37 °C for 18 h, the presence/absence (±) of antimicrobial activity was estimated based on the presence of an inhibition zone inside each quadrant. The assay was performed in duplicate. A negative control comprising 20 mL of sterilized TSB medium concentrated to 1 mL was used for each pathogen test strains following the same protocol.

## Supplementary Information

Supplementary Infomation.

## Data Availability

The partial 16S rRNA gene sequences of the bacterial isolates from cave ice core were deposited in GenBank database under accession numbers MG642093-MG642131, MG642137-MG642141, MG680928, MG680933-MG680934, MH321580-MH321599, MH321601-MH321603, MN577392-MN577395, MN577397, MN577399, MN577401, MN577406, MN577408.
