# Supplementary material for: First report on antibiotic resistance and antimicrobial activity of bacterial isolates from 13,000-year old cave ice core
Source: Sci Rep. 2021 Jan 12;11:514. doi: 10.1038/s41598-020-79754-5 (PMC7804186; doi:10.1038/s41598-020-79754-5)
Supplement: Supplementary file 1 — Supplementary Infomation. [file 41598_2020_79754_MOESM1_ESM.pdf]

SUPPLEMENTARY MATERIAL

First report on antibiotic resistance and antimicrobial activity of bacterial isolates from 13,000-year old cave ice core

Victoria I. Paun[1], Paris Lavin[2,3], Mariana C. Chifiriuc[4], Cristina Purcarea[1]

Supplementary Table S1. Bacterial isolates from Scarisoara cave ice core. ([++, *, **]): strains with identical 16S rRNA gene sequence but different growth temperatures and/or antibiotics susceptibility (Supplementary Table S3). ([ce]): cold environments

Phylum	Sample code	Best Match	Identity % (bp coverage)	Best Match Origin	Growth temperature (°C)
Actinobacteria	SC51B.1[MG680928]	*Aeromicrobium fastidiosum 10552 [NR_119352.1]*	99 (754)	Plant, UK	4-30
	SC97D.2[MH321587]	*Aeromicrobium panaciterrae Gsoil 161 [NR_041382.1]*	94 (1266)	Ginseng soil, South Korea	4-30
	SC86D.2[MG642125]	*Agreia pratensis P229/10 [NR_025460.1]*	100 (805)	Grass, Germany	4-30
	SC41AB.3[MN577401]	*Arthrobacter agilis 20550 [NR_026198.1]*	96 (1138)	Waste	4-20
	SC86E.1[MG642109]	*Arthrobacter alpinus S6-3 [NR_117254.1]*	99 (942)	Alpine soil, Austria[ce]	4-25
	SC21C.1[MG642119]	*Arthrobacter psychrochitiniphilus GP3 [NR_104702.1]*	100 (535)	Penguin guano, Antarctica[ce]	4-25
	SC7AB.1[MH321581]	*Arthrobacter psychrolactophilus B7 [NR_025003.1]*	97 (1180)	Soil	4-25
	SC86D.5[MG642141]	*Conyzicola lurida HWE2-01 [NR_134011.1]*	99 (858)	Horseweed root, South Korea	4-25
	SC21E.1[MH321582]	*Cryobacterium levicorallinum Hh34 [NR_109365.1]*	99 (1229)	China No.1 Glacier[ce]	4-15
	SC14E.1[MH321603]	*Cryobacterium arcticum SK1 [NR_108605.1]*	90 (1217)	Soil, Greenland[ce]	4-25
	SC83A.1[MG642097]	*Cryobacterium flavum Hh8 [NR_104993.1]*	100 (961)	China No.1 Glacier[ce]	4-37
	SC61A.5B[MN577393]	*Dietzia natronolimnaea 44860 [NR_116683.1]*	96 (1268)	East African soda lake	4-37
	SC21E.3[MG642116]	*Glaciihabitans tibetensis MP203 [NR_133754.1]*	99 (537)	Midui Glacier,China[ce]	4-25
	SC7AB.2[MG642137]	*Leifsonia antarctica SPC-20 [NR_042688.1]*	99 (609)	Core sediments, Antarctic Ocean[ce]	4-25
	SC1A.5[MN577392]	*Microbacterium ginsengiterrae DCY37 [NR_116483.1]*	98 (1173)	Ginseng soil, South Korea	4-30
	SC93A.1[MG642127]*	*Microbacterium hydrocarbonoxydans BNP48 [NR_042263.1]*	99 (730)	Oil-contaminated soil, Germany	4-37
	SC8A.4[MG642114]*		99 (725)		4-37
	SC56A.3[MG642140]*		99 (700)		4-37
	SC80A.1[MG642111]*		99 (647)		4-37
	SC1A.3[MG642094]*		99 (816)		4-37
	SC18B.1[MG642103]*		99 (907)		4-37
	SC86D.1[MH321583]	*Microbacterium lacus A5E-52 [NR_041563.1]*	99 (1210)	Lake sediment, Japan	4-25
	SC70A.1[MG642106]	*Microbacterium paraoxydans CF36 [NR_025548.1]*	99 (746)	Human blood	4-30
	SC51B.2[MG642121]**	*Microbacterium pygmaeum KV-490 [NR_041406.1]*	99 (612)	Soil, Japan	4-30
	SC65A.2[MG642099]**		99 (951)		4-37
	SC14F.2[MG642118]**		99 (648)		4-30

	SC83A.2[MG642120]**		99 (539)		4-37
	SC51A.2[MG642100]	*Mycetocola manganoxydans MB1-14 [NR_117467.1]*	99 (870)	Desert sand, China	4-25
	SC86C.2[MG642124]	*Nocardioides terrigena DS-17 [NR_044185.1]*	97 (679)	Soil, Dokdo island	4-30
	SC70B.1[MH321584]	*Pseudarthrobacter oxydans 20119 [NR_026236.1]*	97 (1180)	Air	4-25
	SC86E.4[MN577395]	*Pseudarthrobacter polychromogenes 20136 [NR_026192.1]*	95 (1168)	Air	4-30
	SC80A.3[MN577397]	*Salinibacterium xinjiangense 0543 [NR_043893.1]*	97 (1126)	China No.1 Glacier[ce]	4-15
Proteobacteria	SC86E.5[MG642110]	*Brevundimonas bullata 13290 [NR_113611.1]*	99 (758)	Soil	4-25
	SC14F.3[MH321586]	*Brevundimonas intermedia 15262 [NR_041966.1]*	98 (1171)	Pond water	4-25
	SC14E.3[MG642117]	*Brevundimonas lenta DS-18 [NR_044186.1]*	99 (823)	Soil, South Korea	4-25
	SC21C.2[MG642131]	*Brevundimonas staleyi FWC43 [NR_114710.1]*	98 (699)	Sludge, Canada	4-25
	SC8A.3[MG642113]	*Candidimonas bauzanensis BZ59 [NR_108569.1]*	98 (807)	Hydrocarbon-contaminated soil, Italy	4-30
	SC61A.5[MH321601]	*Caulobacter henricii 15253 [NR_025319.1]*	89 (940)	Freshwater	4-30
	SC71.5[MN577406]	*Delftia acidovorans 14950 [NR_113708.1]*	95 (1181)	Acetamide enriched soil, Netherlands	4-30
	SC41AB.2[MG642139]	*Eoetvoesia caeni PB3-7B [NR_125538.1]*	98 (640)	Activated sludge, Hungary	10-25
	SC71.3[MH321589]	*Paralcaligenes ginsengisoli DCY104 [NR_148318.1]*	94 (1029)	Ginseng soil, South Korea	4-30
	SC18B.3[MH321588]	*Paralcaligenes ureilyticus GR24-5 [NR_116812.1]*	91 (1132)	Ginseng soil, South Korea	4-30
	SC71.1B[MN577394]	*Phenylobacterium haematophilum [NR_041991.1]*	96 (1145)	Human blood, Sweden	10-25
	SC71.2[MH321598]	*Phyllobacterium loti S658 [NR_133818.1]*	91 (997)	Lotus sp., Uruguay	10-30
	SC14F.1[MH321585]	*Phyllobacterium trifolii PETP02 [NR_043193.1]*	98 (1299)	*Trifolium pratense*, Spain	4-25
	SC97A.1[MG642101]	*Pseudomonas brenneri 97-391 [NR_025103.1]*	99 (808)	Natural mineral water, France	4-30
	SC97A.2[MG642102]	*Pseudomonas grimontii 97-514 [NR_025102.1]*	99 (857)	Natural mineral water, France	4-30
	SC80A.2[MN577408]	*Psychrobacter glaciei BIc20019 [NR_148850.1]*	98 (1032)	Ice core, Svalbard[ce]	4-25
	SC18B.1[MH321602]	*Pusillimonas harenae B201 [NR_108541.1]*	94 (970)	Beach sediment, South Korea	4-30
	SC7AB.4[MG642138]	*Rhizorhabdus argentea SP1 [NR_133845.1]*	99 (789)	Soil, Spain	4-25
	SC61B.1[MG642130]	*Sphingomonas oligophenolica S213 [NR_024685.1]*	99 (619)	Paddy soil, Japan	4-30
Firmicutes	SC93A.3[MH321592]	*Bacillus safensis FO-36b [NR_041794.1]*	99 (1225)	Spacecraft assembly room, USA	10-37
	SC97D.6[MH321594]	*Bacillus safensis 100820 [NR_113945.1]*	98 (1269)	Clean room air, USA	10-37
	SC8A.7[MH321599]	*Bacillus thuringiensis 10792 [NR_114581.1]*	96 (1262)	Mediterranean flour moth	10-37
	SC8A.1[MH321590]	*Bacillus thuringiensis 12077 [NR_043403.1]*	96 (1252)	Mediterranean flour moth	10-37
	SC86E.3[MH321591]	*Bacillus toyonensis BCT-7112 [NR_121761.1]*	100 (1171)	Soil, Japan	10-37
	SC97D.1[MH321593]	*Paenibacillus amylolyticus NRS-290 [NR_025882.1]*	98 (1215)	Soil, USA	10-37
	SC80A.5[MG642128]	*Paenisporosarcina macmurdoensis CMS 21w [NR_025573.1]*	99 (591)	Cyanobacterial mat, Antarctica[ce]	4-37
	SC21C.2[MG680933]	*Sporosarcina globispora 16082 [NR_113837.1]*	100 (637)	Soil	4-25
	SC21C.3[MG680934]	*Sporosarcina psychrophila W16A [NR_036942.1]*	94 (537)	Soil	4-25

Bacteroidetes	SC93B.1[MN577399]	*Chryseobacterium molle DW3 [NR_042160.1]*	96 (1086)	biofilm of beer-bottling plant, Germany	10-37
	SC86C.1[MG642123]++	*Chryseobacterium hominis NF802 [NR_042517.2]*	99 (840)	Blood, Belgian patient	4-37
	SC89A.1[MG642126]++		99 (904)		4-37
	SC70A.2[MG642107]++		99 (741)		4-37
	SC1A.2[MG642093]	*Flavobacterium glaciei 0499 [NR_043891.1]*	97 (852)	Soil, China No.1 Glacier[ce]	4-20
	SC21E.2[MG642115]	*Mucilaginibacter phyllosphaerae PP-F2F-G21 [NR_152043.1]*	97 (670)	Phyllosphere of Galium album	4-30
	SC1A.4[MG642095]	*Pedobacter bambusae THG-G118 [NR_148294.1]*	99 (834)	Bamboo soil, South Korea	4-28
	SC1A.1[MH321580]	*Pedobacter steynii WB 2.3-45 [NR_042605.1]*	96 (1242)	Hardwater rivulet, Germany	4-25

Supplementary Table S2. Distribution of bacterial isolates along Scarisoara cave ice core. The age of cave ice samples used for bacterial isolation was determined as calibrated years before present (cal BP) (Paun et al., 2019).

**Number of strains**	**Strain code**	**Age (cal BP)**
5	SC1A.1, SC1A.2, SC1A.3, SC1A.4, SC1A.5	92 ±26
3	SC7AB.1, SC7AB.2, SC7AB.4	430 ±14
4	SC8A.1, SC8A.3, SC8A.4, SC8A.7	475 ±17
5	SC14E.1, SC14E.3, SC14F.1, SC14F.2, SC14F.3	703 ±23
3	SC18B.1, SC18B.1, SC18B.3	953 ±22
7	SC21C.1, SC21C.2, SC21C.2, SC21C.3, SC21E.1, SC21E.2, SC21E.3	1,124 ±31
2	SC41AB.2, SC41AB.3	2,671 ±36
3	SC51A.2, SC51B.1, SC51B.2	3,937 ±66
1	SC56A.3	4,715 ±46
3	SC61A.5, SC61A.5B, SC61B.1	4,991 ±25
1	SC65A.2	5,335 ±54
3	SC70A.1, SC70A.2, SC70B.1	7,124 ±143
4	SC71.1B, SC71.2, SC71.3, SC71.5	7,382 ±39
4	SC80A.1, SC80A.2, SC80A.3, SC80A.5	8,674 ±59
2	SC83A.1, SC83A.2	9,248 ±111
9	SC86C.1, SC86C.2, SC86D.1, SC86D.2, SC86D.5, SC86E.1, SC86E.3, SC86E.4, SC86E.5	10,022 ±140
1	SC89A.1	11,122 ±118
3	SC93A.1, SC93A.3, SC93B.1	12,053 ±92
5	SC97A.1, SC97A.2, SC97D.1, SC97D.2, SC97D.6	13,223 ±118

Supplementary Table S3. Antimicrobial susceptibility profile, for the 68 bacterial isolated strains. Light green – Susceptible, standard dose; Blue – Resistant.

Phylum	Isolated strains	Antibiotics																											
		penicillins		cephalosporins						carbapenems	fluoroquinolones		macrolides		aminoglycosides			lincosamides		metronidazole	fatty acyls	coumarin glycosides	chloramphenicol	nitrofurantoin	rifampin	sulfonamide compounds	tetracyclines	trimethoprim/sulfonamides	glycopeptides
		**AMP**	**CAR**	**CAZ**	**CFM**	**CTX**	**CPO**	**KF**	**CPD**	**IPM**	**CIP**	**NA**	**CLR**	**E**	**CN**	**SH**	**S**	**DA**	**MY**	**MTZ**	**MUP**	**NV**	**C**	**F**	**RD**	**S3**	**TE**	**W**	**VA**
**Actinobacteria**	**SC51B.1**	S	S	R	R	R	S	R	R	S	R	R	R	S	R	R	R	R	R	R	R	S	S	R	S	R	S	R	R
	**SC97D.2**	S	R	R	R	R	R	R	R	S	R	R	R	R	R	R	R	R	R	R	R	S	S	R	S	R	S	R	S
	**SC86D.2**	S	R	R	R	S	S	S	S	S	S	R	R	S	R	R	R	R	S	R	R	S	S	R	S	S	S	S	S
	**SC41AB.3**	S	S	R	R	S	S	S	S	S	R	R	S	S	R	R	S	R	R	R	R	S	S	R	S	R	S	S	S
	**SC86E.1**	S	S	R	R	R	S	R	R	S	R	R	R	R	R	R	R	R	R	R	R	R	S	R	S	R	S	R	R
	**SC21C.1**	S	S	R	R	R	R	S	R	S	R	R	S	S	R	R	R	R	S	R	R	S	S	R	S	R	S	R	S
	**SC7AB.1**	S	S	S	R	S	S	S	S	S	S	R	S	S	S	S	S	S	S	S	S	S	S	S	S	S	S	S	S
	**SC86D.5**	S	S	R	R	S	S	R	R	S	S	R	S	S	R	R	R	R	S	R	R	S	S	R	S	S	S	S	S
	**SC21E.1**	S	S	R	R	R	R	R	R	S	S	R	S	R	R	R	R	R	R	R	R	R	S	R	S	S	R	S	R
	**SC14E.1**	R	R	R	R	R	R	R	R	S	S	R	R	R	R	R	R	R	R	R	R	S	S	R	R	R	S	R	R
	**SC83A.1**	S	S	S	R	R	R	S	S	S	S	R	R	S	R	R	S	S	S	R	R	S	S	R	S	R	S	S	S
	**SC61A.5B**	R	R	R	R	R	R	R	R	R	S	R	R	R	R	R	R	R	R	R	R	R	R	R	R	R	R	R	R
	**SC21E.3**	S	S	S	R	S	S	S	S	S	S	R	S	S	S	R	S	S	S	R	S	S	S	S	S	R	S	R	S
	**SC7AB.2**	S	S	R	R	R	R	S	R	S	S	R	R	S	R	R	S	R	S	R	R	S	S	R	S	S	S	S	S
	**SC1A.5**	S	R	R	R	R	R	R	R	S	S	R	S	S	R	R	R	R	R	R	R	R	R	R	S	R	S	S	S
	**SC93A.1**	R	R	R	R	R	R	R	R	S	S	R	R	R	R	R	R	R	R	R	R	R	R	R	S	R	R	S	S
	**SC8A.4**	R	R	R	R	S	R	R	R	S	S	R	S	S	R	R	R	R	R	R	R	R	R	R	S	R	R	S	S
	**SC56A.3**	R	R	R	R	S	R	R	R	S	S	R	S	S	R	R	R	R	R	R	R	R	R	R	S	R	R	S	S
	**SC80A.1**	R	R	R	R	R	R	R	R	S	R	R	R	R	R	R	R	R	R	R	R	R	R	R	S	R	R	S	R
	**SC1A.3**	R	R	R	R	R	R	R	S	S	R	R	S	S	R	R	R	R	R	R	R	R	R	R	R	R	R	S	R
	**SC18B.1**	S	S	R	R	S	S	S	R	S	S	R	S	S	R	R	R	R	S	R	R	S	S	S	S	R	S	R	S
	**SC86D.1**	R	R	R	R	R	R	R	R	R	S	R	R	R	R	R	R	R	R	R	R	S	R	R	S	S	S	R	S
	**SC70A.1**	S	S	R	R	S	S	S	R	S	S	R	R	R	R	R	R	R	S	R	R	S	S	S	S	R	S	R	S
	**SC51B.2**	S	S	R	R	S	R	S	R	S	S	R	R	S	R	R	S	R	S	R	R	S	S	S	S	R	S	R	S
	**SC65A.2**	S	S	R	R	R	S	R	R	S	R	R	S	S	R	R	R	S	R	R	R	S	S	R	S	R	S	R	S
	**SC14F.2**	S	R	R	R	R	R	S	R	S	S	R	R	S	R	R	R	R	R	R	R	R	S	R	S	R	S	S	S

	SC83A.2	R	R	R	R	R	R	R	R	S	R	R	R	R	R	R	R	R	R	R	R	R	R	R	R	R	R	R	R
	SC51A.2	S	S	R	R	S	R	S	R	S	S	R	S	S	R	R	S	R	S	S	R	S	S	S	S	S	S	S	S
	SC86C.2	S	R	R	R	R	R	S	R	S	S	R	S	S	R	R	S	R	R	R	R	R	S	R	S	R	S	R	S
	SC70B.1	R	S	R	R	R	R	R	R	R	R	R	R	R	R	R	R	R	R	R	R	R	R	R	R	R	R	R	R
	SC86E.4	S	S	R	R	R	R	S	R	S	R	R	S	S	R	R	R	R	R	R	R	S	S	R	S	S	S	R	S
	SC80A.3	S	S	R	R	R	R	S	S	S	S	R	R	S	R	R	R	R	S	R	R	S	S	R	S	R	S	R	S
Firmicutes	SC93A.3	S	S	R	R	R	R	S	R	S	S	R	S	S	S	R	S	R	R	R	R	R	S	S	R	S	S	S	S
	SC97D.6	S	S	R	R	R	R	S	R	S	R	S	S	S	S	R	R	R	R	S	R	R	R	S	R	S	S	S	R
	SC8A.	R	R	R	R	R	R	R	R	S	S	S	S	S	R	R	R	R	R	R	R	S	S	S	R	R	S	R	R
	SC8A.1	R	R	R	R	R	R	R	R	S	R	R	S	S	R	R	S	S	R	R	R	S	S	S	R	R	S	R	S
	SC86E.3	R	R	R	R	R	R	R	R	S	S	S	S	S	R	R	R	R	R	R	R	R	S	S	S	R	S	R	S
	SC97D.1	S	S	R	R	R	R	R	R	S	R	S	S	R	S	R	R	R	R	S	R	R	R	S	R	S	S	S	R
	SC80A.5	S	S	R	R	S	S	S	S	S	S	R	S	S	S	S	S	S	R	R	S	S	S	S	S	R	S	S	S
	SC21C.2	S	R	R	R	R	R	R	R	S	S	R	R	R	R	R	R	R	R	R	R	R	R	R	S	R	S	R	R
	SC21C.3	S	S	R	S	S	S	S	S	S	S	R	S	S	S	R	S	R	S	R	S	S	S	S	S	R	S	S	S
Bacteroidetes	SC93B.1	R	R	R	R	R	R	R	R	R	R	S	R	R	R	R	R	R	R	R	R	R	S	R	S	R	S	R	R
	SC86C.1	R	R	R	R	R	R	R	R	R	S	R	S	R	R	R	R	R	R	R	R	S	R	R	S	R	S	S	R
	SC89A.1	R	R	R	R	S	S	R	R	R	S	S	R	R	R	R	R	R	R	R	R	S	R	S	S	R	S	S	R
	SC70A.2	R	R	R	R	R	R	R	R	R	S	S	R	R	R	R	R	R	R	R	R	S	R	S	S	R	S	R	R
	SC1A.2	R	R	R	R	R	R	R	R	S	R	R	R	R	R	R	R	R	R	R	R	R	R	R	S	R	S	R	R
	SC21E.2	R	R	R	R	R	R	R	R	R	R	R	R	R	R	R	R	R	R	R	R	R	R	R	R	R	S	R	R
	SC1A.4	R	S	R	R	R	R	R	R	S	R	R	R	S	R	R	R	R	S	R	S	S	S	S	S	S	S	S	R
	SC1A.1	R	R	R	R	R	R	R	R	S	R	R	R	R	R	R	R	R	R	R	R	R	S	R	R	S	S	S	R
Proteobacteria	SC86E.5	R	R	R	R	R	R	R	R	S	R	R	R	R	R	R	R	R	R	R	R	R	S	R	S	R	S	R	R
	SC14F.3	R	R	R	R	R	R	R	R	S	R	R	R	R	R	R	R	R	R	R	R	R	S	R	R	R	S	R	S
	SC14E.3	R	R	R	R	R	R	R	R	S	R	R	R	R	R	R	R	R	R	R	R	S	R	R	S	S	S	R	S
	SC21C.2	S	S	R	R	R	S	R	R	S	R	R	R	R	R	R	R	R	R	R	R	S	S	R	S	R	S	R	R
	SC8A.3	R	R	R	S	S	R	R	R	R	R	S	R	R	R	R	R	R	R	R	R	R	R	R	R	R	R	R	R
	SC61A.5	S	S	R	R	R	R	R	R	S	R	R	R	S	R	R	R	S	R	R	R	S	S	R	S	R	S	R	S
	SC71.5	R	R	R	R	R	R	R	R	S	S	R	R	R	R	R	R	R	R	R	R	R	R	R	R	R	R	R	R
	SC41AB.2	S	R	R	R	S	R	S	R	S	S	R	S	S	R	R	S	R	S	R	R	S	R	S	S	R	S	R	S
	SC71.3	R	R	R	R	R	R	R	R	S	S	R	R	R	R	R	R	R	R	R	R	R	R	R	R	R	S	R	R
	SC18B.3	R	R	R	R	R	R	R	R	S	R	R	R	R	R	R	R	R	R	R	R	R	R	R	R	R	R	R	R
	SC71.1B	R	R	R	R	R	R	R	R	R	R	R	R	R	R	R	R	R	R	R	R	R	R	R	S	R	S	R	R
	SC71.2	R	R	R	R	R	R	R	R	R	R	R	R	R	R	R	R	R	R	R	R	R	R	R	S	R	S	R	R
	SC14F.	R	R	R	R	R	R	R	R	S	R	R	R	R	R	R	R	R	R	R	R	R	R	R	S	R	S	R	R
	SC97A.1	R	R	R	R	R	R	R	R	R	R	R	R	R	R	R	R	R	R	R	R	R	R	R	R	R	R	R	R
	SC97A.2	R	R	R	R	R	R	R	R	R	R	R	R	R	R	R	R	R	R	R	R	R	R	R	R	R	R	R	R
	SC80A.2	S	S	R	R	S	S	R	S	S	S	S	R	R	R	R	S	R	R	R	R	R	S	R	R	S	S	R	R
	SC18B.1	R	R	R	R	S	R	R	R	R	R	R	S	R	R	R	R	R	R	R	R	R	R	S	R	R	R	R	R
	SC7AB.4	S	S	R	R	R	R	R	R	S	R	R	S	R	R	R	R	R	R	R	R	S	S	R	S	R	S	R	R
	SC61B.1	R	R	R	R	R	R	R	R	S	R	R	R	R	R	R	R	R	R	S	R	R	R	R	S	S	S	S	S

Supplementary Table S4. Bacterial pathogen strains used for testing the antimicrobial potential of bacterial strains isolated from Scarisoara ice cave. Clinical isolated strains were from the Research Institute of The University of Bucharest Microbial Collection.

**Bacterial pathogens**
Staphylococcus aureus ATCC 25923, G+ (Thermo Scientific, USA)
Escherichia coli ATCC 25922, G- (Thermo Scientific, USA)
Clinical isolated pathogens
Enterobacter cloacae 19069 ONE2, G-
Enterobacter cloacae 19069 ONE3, G-
Enterobacter asburiae 19069 ONE1, G-
Pseudomonas CN11, G-
Pseudomonas aeruginosa 19053 CNE5, G-
Pseudomonas aeruginosa 19053 CNE6, G-
MRSA 388, G+
MRSA 19081 F1, G+
MRSA 19081 S1, G+
Klebsiella 8, G-
Klebsiella 19094 CK1, G-
Klebsiella 19094 CK2, G-
Klebsiella 19094 CK3, G-
Acinetobacter 19047 ENE4, G-
Acinetobacter 19047 CNE5, G-
Acinetobacter 19047 CNE3, G-
Acinetobacter 18032 C3, G-
Enterococcus falcium 19040 E1, G+
Enterococcus falcium 19040 E2, G+
Enterococcus falcium 19040 E3, G+
